# The effects of climate change on EO/IR propagation using CMIP6 global atmospheric forecasting simulations

**DOI:** 10.1038/s41598-025-99306-z

**Published:** 2025-04-25

**Authors:** Parker Coye, Adam Willitsford

**Affiliations:** Johns Hopkins University Applied Physics Lab, 11100 Johns Hopkins Rd, Laurel, MD 20723 USA

**Keywords:** Climate change, EO/IR, Transmission, MODTRAN, CMIP6, Atmospheric science, Atmospheric optics

## Abstract

Climate change-driven atmospheric effects are of particular concern to those who operate electro-optic and infrared (EO/IR) sensors, as atmospheric constituents such as water vapor, carbon dioxide, and aerosols drive the absorption and scattering effects necessary to characterize deployed optical system performance. Current models of EO/IR propagation are fed by statistics built off the historical state of the atmosphere by utilizing ground based observations, satellite data, or reanalysis datasets. Such methods are effective at characterizing EO/IR propagation for historical time periods, but do little to inform decisions related to future sensor deployment. This work utilizes future projections of atmospheric variables from the Coupled Model Intercomparison Project (CMIP6), an international collection of climate models, to characterize atmospheric transmittance, a metric closely tied to EO/IR performance. Analysis of regional transmittance (particularly in the long-wave infrared) reveals drops by as much as 20% from 2015-2100 for a path as short as 2 km - this is nearly a doubling of the band averaged extinction coefficient.

## Introduction

Central to accurately characterizing the performance of terrestrial Electro-Optic/Infrared (EO/IR) sensors is having an understanding of the atmospheric conditions in which they operate. Current evaluations for EO/IR sensor performance as well as laser propagation rely on past and present measurements^1–3^as well as models^4,5^ of the atmospheric conditions. These historical and present-day datasets provide context for evolving atmospheric variability which is being accelerated by the effects of climate change in the form of increases in extreme rain events, atmospheric temperature, carbon dioxide, specific humidity, and $\mathrm {PM_{2.5}}$(particulate matter with a diameter of 2.5 microns or less, corresponding to poor air quality) to name a few^6–8^.

The severity of some of these changes is highly regional in nature; for example, developing nations are more reliant on fossil fuels and generally are subject to fewer air quality restrictions, while both the United States (US) and European Union (EU) have been working to improve air quality via adoption of clean air policy and greener technology. In the United States, the Clean Air Act passed in 1967 and monitored by the Environmental Protection Agency has contributed significantly to a reduction in particulates which drives improvements in air quality. The US has monitored these changes via the IMPROVE (The Interagency Monitoring of Protected Visual Environments)^9^program, which was started in 1988 and has produced reports showing improvement in air quality and visibility. Similarly, the EU has been evaluating air quality since the 1970 s under the European Monitoring and Evaluation Programme (EMEP)^10^. In addition to regional pollution challenges due to differences in industry regulation, meteorological extremes such as heat waves, floods, and droughts are projected to become increasingly commonplace^6^.

The combined effects of these atmospheric changes will have detrimental effects on optical propagation. In the worst case EO/IR imaging, detection, and identification ranges will decrease, free-space optical (laser) communications will see decreased link availability, and decreased range performance. Both the Department of Defense^11^and the civilian astronomy communities^12,13^ have shared concerns as climate change will challenge future EO/IR sensors and their applications.

Several applications where EO/IR remote sensed imagery is being further leveraged to aid in decision making and real-time evaluations include: crop health monitoring and food security^14–16^, animal migration and wildlife protection^17–19^, forest fire detection^20,21^, oil spill detection^22,23^, driver assistance systems and highway traffic monitoring^24–26^, and power line inspections^27,28^. In each instance both elevated platforms (airborne/spaceborne) as well as surface level imagery is utilized for detailed decision support. The use of surface level infrared imagery for the detection of migrating whales has been shown over horizontal paths exceeding 1 km, however, in times of reduced visibility (fog) the ability to detect the whales become more challenging^18^. This decrease in detection is analogous to the threat posed by increased atmospheric water vapor resulting from climate change.

Here we specifically evaluate changes in surface level horizontal path transmittance by using MODerate Resolution TRANsmittance (MODTRAN)^29^, seeded with future atmospheric predictions from the Coupled Model Intercomparison Project Phase 6 (CMIP6)^30^. Leveraging the large CMIP6 dataset to build statistics on EO/IR propagation enables evaluation of expected transmittance changes due to atmospheric climate change. In particular we look closely at changes in the Long-wave infrared (8.0–12.0 $\mu$m) transmittance as increases in atmospheric water vapor increase path losses due to the high absorbance of water in this band. This work furthers the goal of operationalizing climate models, prioritizing information most impactful to decision-makers^31^.

## Electro-optic and infrared (EO/IR) propagation overview

Atmospheric transmittance and path/background radiance drive one’s ability to detect and analyze targets using EO/IR sensors, as susceptibility to transmittance losses due to aerosol scattering can often be a significant driver of performance. Atmospheric transmittance is affected differently depending on the wavelength of interest but in general, major atmospheric performance drivers include particulate matter (aerosols), water (vapor/rain), carbon dioxide, as well as other gases that can absorb and scatter the light propagating from target to sensor. A practiced method for constructing operational metrics due to meteorological variability is via statistical aggregation for historical time periods^32^. Although this method is effective for predicting EO/IR sensor performance for the present day, systems that are currently being designed need to be operational for decades to come. Therefore it becomes necessary to utilize decadal forecasts of atmospheric variables to characterize EO/IR sensor performance in our rapidly evolving environment.

## CMIP6 overview

CMIP6 is an international collection of climate models considered to be one of the standards in large scale simulations of Earth processes^30^. The collection of models from CMIP6 chosen for this work are contained in a sub-project (Model Intercomparison Project, MIP) called “ScenarioMIP”, which lays out forecasts for a wide variety of oceanic and atmospheric variables under different “Shared Socioeconomic Pathways (SSPs)”^33^. There are five SSPs in ScenarioMIP: SSP1 - Sustainability, SSP2 - Middle of the Road, SSP3 - Regional Rivalry, SSP4 - Inequality, SSP5 - Fossil-fuel Development^34^. These SSPs characterize the climate trajectories the Earth could take, dependent on socioeconomic changes i.e. climate policy and projected advances in technology which impact greenhouse gas emissions and levels of radiative forcing. The two SSPs chosen for comparison in this study are SSP1-2.6 and SSP5-8.5, which represent “best case” and “worst case” scenario climate trajectories, respectively. SSP1-2.6 (best case) corresponds with radiative forcing levels of 2.6 $W/m^2$in the year 2100 (corresponding to Representative Concentration Pathway, RCP, 2.6 in CMIP5^35^) and is characterized by a society taking steps towards sustainability. SSP5-8.5 (worst case) corresponds with radiative forcing levels of 8.5 $W/m^2$ in the year 2100 (corresponding to RCP 8.5) and is characterized by a society leaning into fossil-fueled development. Projections of atmospheric variables decades into the future of course carry inherit uncertainty, due to all sorts of factors such as unknowns in predicted human behavior/societal development, differences in model initialization from measurement uncertainty, and varying physics representations of feedback mechanisms. Although this study only evaluates one model (as explained further below) and uncertainties within that model certainly propagate to our analysis, evaluation of the “best case” and “worst case” scenarios within that model help to bound the future.

The first step to analyzing the temporal evolution of global transmittance due to meteorological and climatological changes in our atmosphere is analysis of the dominant atmospheric variables which effect EO/IR propagation. In order to first inspect the atmospheric trends more broadly before applying the data to EO/IR performance metrics, key atmospheric variables such as surface temperature, pressure, and specific humidity were statistically analyzed via inspection of their cumulative distribution function (CDF). A CDF describes the probability, or effective likelihood of a given value in a dataset to occur. In this case, the $50^{th}$ percentile values were used for a given variable to represent an “average” day.

**Fig. 1:**
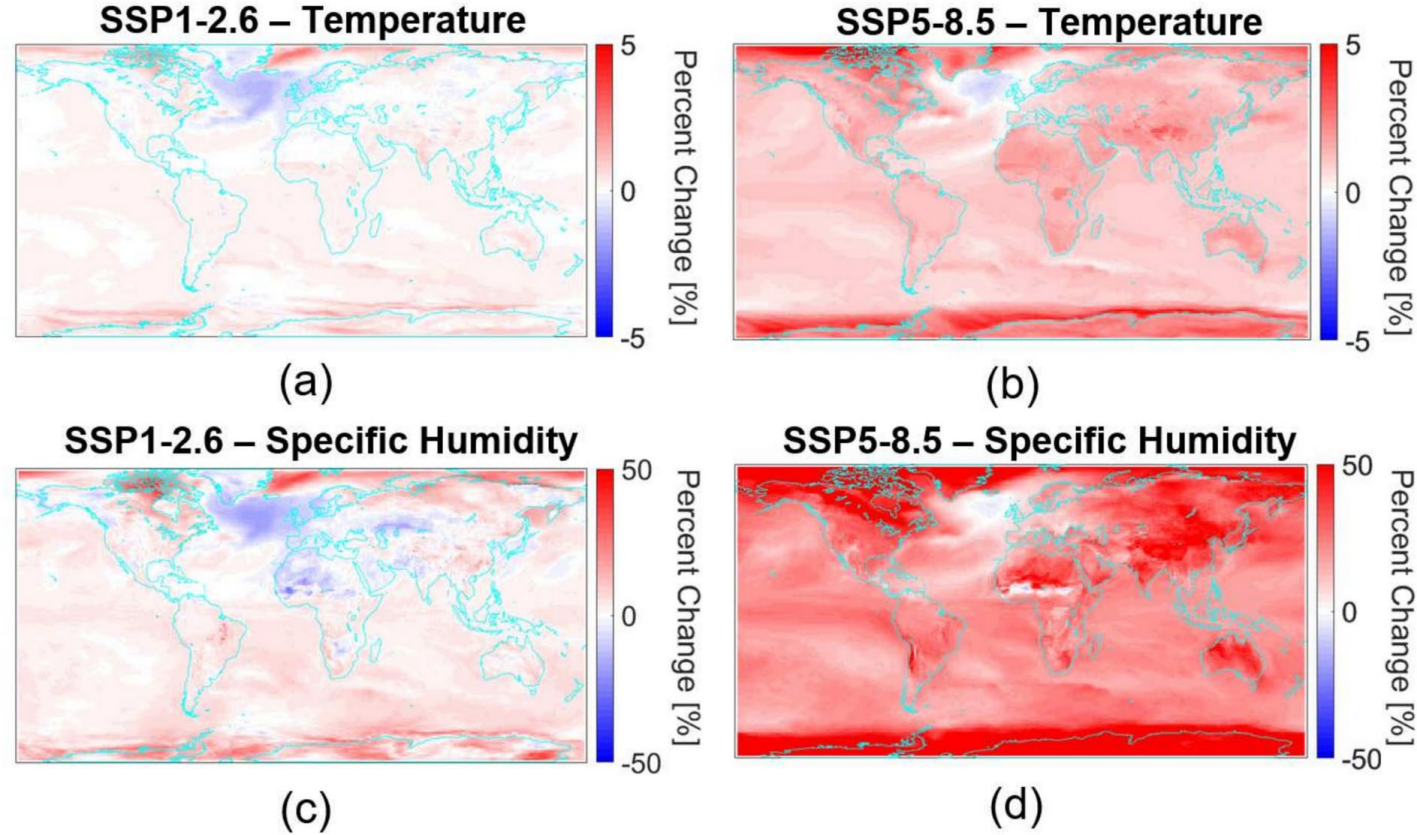
Percent change from 2015 to 2100 in temperature (**a**) (**b**) and specific humidity (**c**) (**d**) for SSP1-2.6 and SSP5-8.5 (MRI-ESM).

As shown in Figure 1, temperature and specific humidity increase nearly everywhere on the globe, with more extreme increases observed under the “worst case scenario” SSP5-8.5. These trends will adversely effect EO/IR propagation in multiple ways, for example the specific humidity. A higher absolute water content means that more energy is absorbed, particularly in the Long-wave Infrared (LWIR), which impacts transmittance.

## Methods

### Atmospheric transmittance

Analysis of atmospheric transmittance was performed via modeling using the MODerate Resolution Transmittance (MODTRAN) model^29,36^. MODTRAN is a radiation transfer and atmospheric propagation model that calculates transmittance (absorption + scattering) as well as path radiance (thermal emission + scattered light) from the ultra-violet to the infrared ($\lambda = 200 nm- 100 \mu m$). MODTRAN can be run by ingesting detailed atmospheric data that represents the line of site geometry of interest via several options including (1) using “standard” atmospheres (e.g., 1976 U.S. Standard), (2) using standard atmospheres modified by additional data (pressure, temperature, humidity, visibility, etc - the method used in this study), and (3) using user supplied full custom atmospheric (e.g., radiosonde) profiles. In each case the constructed atmospheric profiles are leveraged to drive physics-based models of the absorption and scattering properties for the atmospheric constituents. This enables a full radiative transfer (transmittance + radiance) along the line of sight from the observer to the target of interest. To evaluate the effects of climate change on EO/IR sensors we leverage the future projections of temperature, pressure, specific humidity, and $\mathrm {CO_2}$ from CMIP6, however due to conflicting literature (detailed in the next section), we choose to separately address aerosol effects and the resulting MODTRAN visibility input which we discuss in detail below.

### Visibility, aerosol effects and MODTRAN

Visibility and aerosol concentration are tightly coupled phenomena as the scattering effects of aerosols can be strong contributors to transmittance losses in the visible wavelength regime. Here we use the Koschmeider^37^ definition of visibility as the range at which transmittance is $=2\%$ at $\lambda$ = 550 nm. The effects of aerosol scattering and absorption losses within MODTRAN are determined by choice of the aerosol model, specific humidity and visibility input fields. The MODTRAN modeling suite has several built-in aerosol profiles including maritime, urban, rural, and desert. These different models can lead to significantly different impacts on EO/IR propagation, particularly in the visible and short-wave infrared wavelength regimes where loss tends to be dominated by scattering. Aerosol concentrations and species are defined in the internal MODTRAN models; the aerosol number densities and size distributions are scaled using the user provided visibility and specific humidity values.

The CMIP6 dataset includes many different climate variables, and among them are aerosol related information. This aerosol information can vary greatly between observations and datasets as they are extended into the future, as researchers disagree on predicted aerosol concentrations that are significant drivers of visibility^39–43^. A key aspect of uncertainty revolves around particulate matter predictions. Machine learning approaches have been applied using historical data (1980-2019) and have shown promise in certain regions of the world. In addition, they predict future reductions in $PM_{2.5}$over most of the globe^44^. This is corroborated with satellite analysis showing reductions in average global $PM_{2.5}$starting from 2011 mostly due to reductions in China^45^. Conversely, other researchers have shown decreases in visibility (increases in $PM_{2.5}$) in China over the same time period with extinction increasing steadily from 2005-2015^40,42^. This is corroborated with other research^43^ which compared several models of CMIP6 to show many of them predict increases in global $PM_{2.5}$.

Aerosol modeling and prediction is incredibly complicated and challenging because of the relatively short lifetimes for aerosols ($\approx$days)^46^as well as the large variability from anthropogenic sources both temporally (seasonality) and spatially. The IPCC states that changes in emissions of short-lived climate forcers (aerosols) and their effects on cooling remains the largest uncertainty in future climate projections^6^.. These climate models lack the resolution necessary to capture the affects of aerosol growth, entrainment and movement which leads to additional challenges in predicting aerosol cloud interactions^47^..

**Fig. 2:**
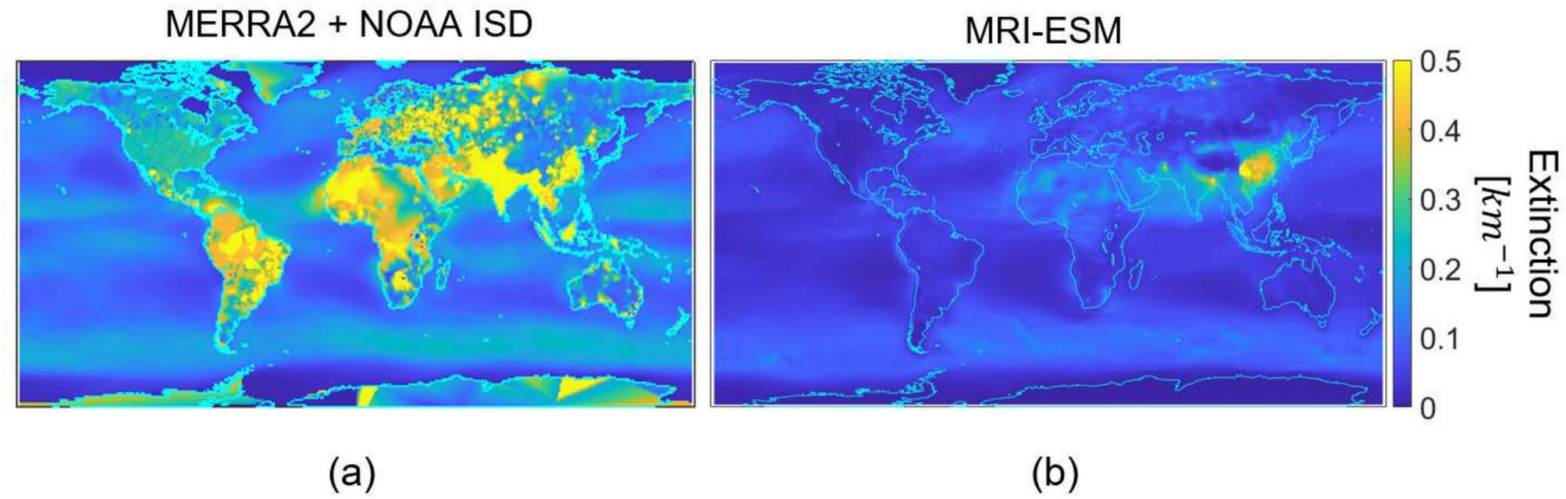
$50^{th}$ percentile aerosol extinction at a wavelength of 550 nm (**a**) derived via ground based observations and reanalysis and (**b**) pulled from a CMIP6 MRI-ESM. Note the strong disagreement.

In addition to the disagreement in the aerosol modeling found by other researchers^39,48–50^, we found that aerosol extinction data provided by CMIP6 in 2018 failed to match our fusion analysis made in the same year. The strongest disagreements were seen in urban areas characterized by high levels of air pollution and can be seen in Figure 2.

Thus due to the large uncertainty in the future projections of aerosols and ultimately visibility, we have chosen to use spatially varying and temporally static aerosol inputs. To set the visibility input field in MODTRAN we leverage statistics built on measured and modeled data, specifically through a fusion of MERRA-2 (reanalysis) data^5^over the ocean and NOAA-NCEI-ISD (ground based observations)^1^ data over land, both from 2018. More details on this fusion can be found in Willitsford, et al.^32^. To perform our analysis, we use the $50^{th}$ percentile values from the 2018 data fusion analysis shown in Figure 2a. If the true trajectory of atmospheric aerosols is increasing into the future, this analysis serves as an upper bound on transmittance and if the true trajectory is decreasing, this analysis is a lower bound.

**Fig. 3:**
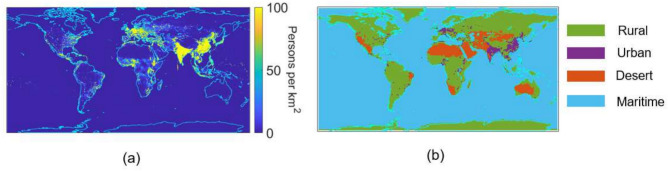
(**a**) Population density via NASA SEDAC in persons per square km (**b**) MODTRAN aerosol model chosen for each location on the globe.

In addition to varying the visibility input in MODTRAN, the built-in aerosol model chosen was also varied depending on the location. For over-water regions we used the maritime aerosol model, for over-land regions we used the rural aerosol model and for regions where population density was greater than 100 persons$\mathrm {/km^{2}}$in the year 2020 (considered moderately populated) we used the urban aerosol model. The population density was determined using the National Aeronautics and Space Administration (NASA) Socioeconomic Data and Applications Center (SEDAC) dataset^51^. The desert locations were chosen via designations from NASA Earth Observatory^52^. Figure 3 shows the population density from the NASA SEDAC dataset for 2020 and the derived aerosol models used for each of the $1.125^{o}\times 1.125^{o}$ grid matched to the CMIP6 datasets.

We construct custom atmospheric input “decks” for MODTRAN using atmospheric data from CMIP6 including temperature, pressure, specific humidity and $\mathrm {CO_2}$concentration all varying both spatially and temporally. This enables running MODTRAN for all areas of the world going out to the year 2100. The input variables were sourced from a single model within CMIP6, specifically using the Meteorological Research Institute Earth System Model version 2.0 (MRI-ESM2)^53^. These variables include surface air temperature(ts), specific humidity (huss), carbon dioxide concentration (co2), and pressure (ps). A filename for surface pressure, for example would be: “ps_Amon_MRI-ESM2-0_ssp126_r2i1p1f1_gn_201501-210012.nc”. Monthly mean data every other January was downloaded at a spatial resolution of $1.125^{o}\times 1.125^{o}$ for both SSP1-2.6 and SSP5-8.5. This relatively sparse temporal sampling was chosen for practicality, as MODTRAN runs are computationally expensive and looking at long time horizons (out to 2100) was prioritized over a high sampling frequency. MRI-ESM2 was chosen as it provided the necessary variables for analysis at the highest horizontal grid resolution ($\approx$125 km) compared to other models which participate in ScenarioMIP. Although it was the highest resolution available, the still fairly large grid size does impose limitations on the analysis as it is quite coarse. MODTRAN has the ability to synthesize high resolution vertical profile data (e.g. radiosonde, pressure dependent numerical weather prediction) which for is required for non-horizontal lines of sight such as satellite look geometries. In our case we are modeling a 2 km horizontal path at the surface and as such we only require the surface level atmospheric inputs from CMIP6. In addition, our path is quite short at 2 km and when modeled within MODTRAN it is assumed to be horizontally uniform and enables the calculation of surface level path transmittance for our line-of-sight. This approach enables an accurate snapshot of horizontal transmittance^32^ within a grid cell as well as global comparisons both spatially and temporally.

**Fig. 4:**
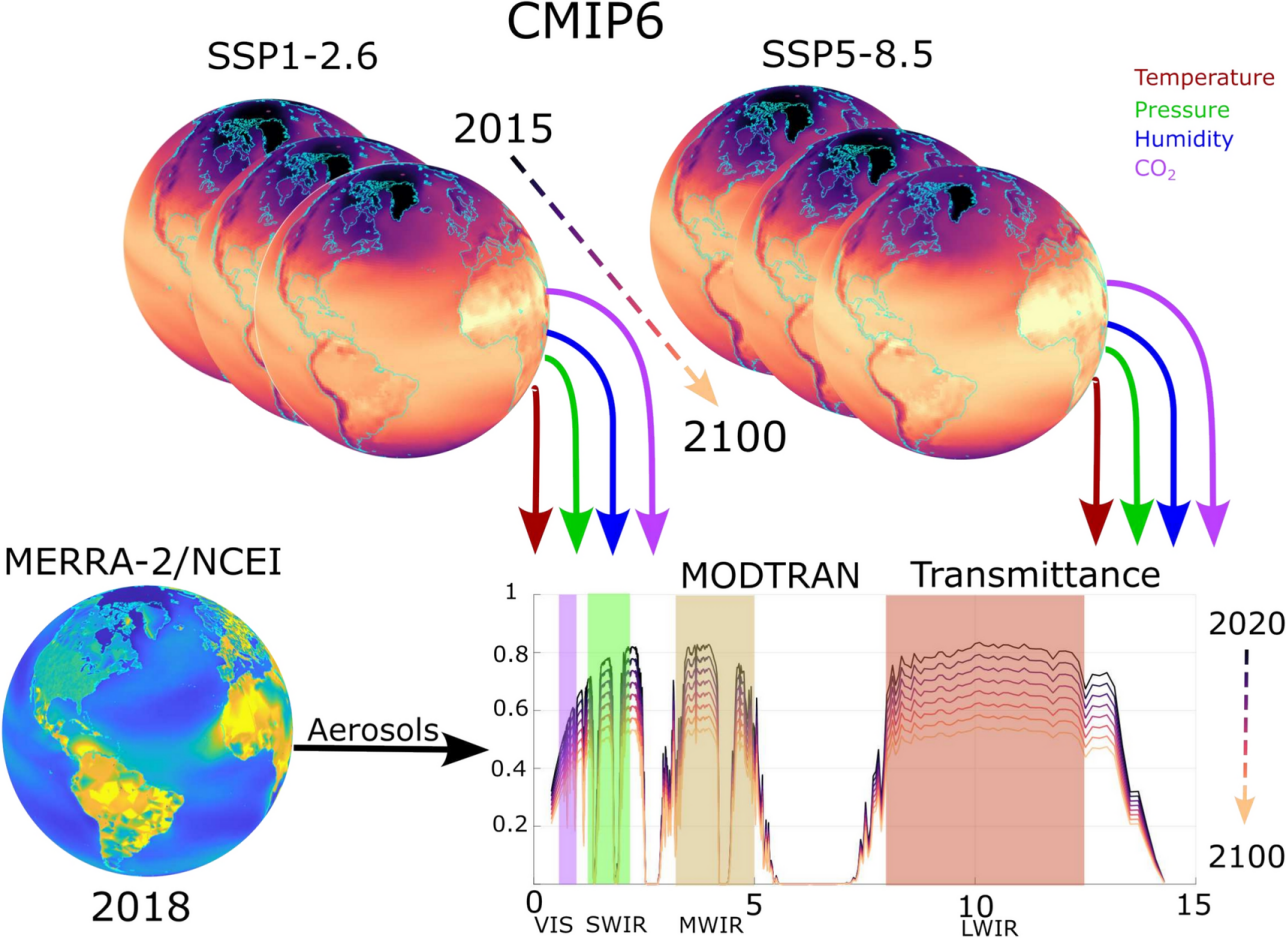
Future atmospheric projections from CMIP6 using SSP1-2.6 (best-case) and SSP5-8.5 (worst-case) from 2015-2100 are coupled with MERRA-2 and NCEI atmospheric aerosol and visibility information to drive MODTRAN atmospheric transmittance analysis. We analyzed four major EO/IR bands of interest including visible (VIS), short-wave infrared (SWIR), mid-wave infrared (MWIR), and longwave infrared (LWIR).

For methodological simplicity, this single model was used for analysis as opposed to an ensemble, therefore the uncertainties associated with its predictions also exist as limitations for our analysis^53^. A visual summary of the data inputs from CMIP6 and the process of integrating with MODTRAN is shown in Figure 4.

**Fig. 5:**
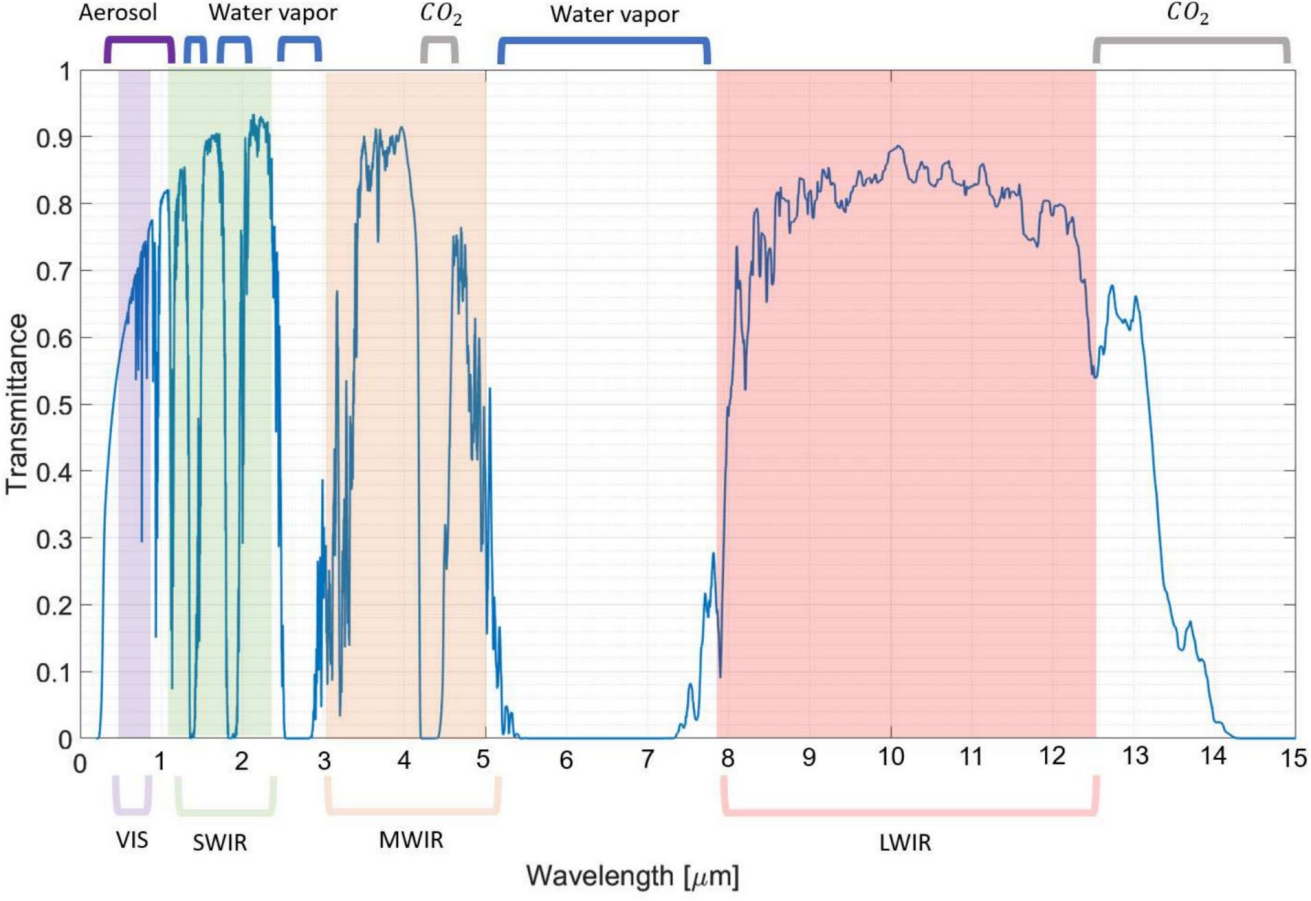
The four EO/IR bands evaluated are shown above along with a typical transmittance vs wavelength plot from MODTRAN. MODTRAN was run for a 2 km horizontal path with the 1976 U.S. Standard atmosphere, rural aerosols and 16 km (10 mi.) visibility.

The meteorological and aerosol variables are inputs into MODTRAN, which uses first principles radiative transfer theory to calculate atmospheric transmittance along the chosen line-of-sight geometry. For this analysis, MODTRAN was run in transmittance mode at the surface with a 2 km horizontal path for four example wavelength regimes of interest: visible [0.4–0.7 $\mu$m]; shortwave infrared (SWIR) [0.9–1.7 $\mu$m], mid-wave infrared (MWIR) [3.0–5.0 $\mu$m], and long-wave infrared (LWIR) [8.0–12.0 $\mu$m]. The wavelength dependent transmittance from MODTRAN was used to calculate a band-averaged transmittance in each of the four bands of interest. This enabled global evaluation of the transmittance as a function of time from 2015-2100. Shown in Figure 5 are the four EO/IR bands on a typical horizontal path transmittance vs. wavelength plot. Major atmospheric performance drivers such as aerosol scattering loss, water vapor concentration, and $\mathrm {CO_2}$ are labeled in wavelength regions where they strongly affect transmittance. Note that the four bands are generally separated by strong atmospheric absorption features.

## Results

### Results - atmospheric transmittance

**Fig. 6:**
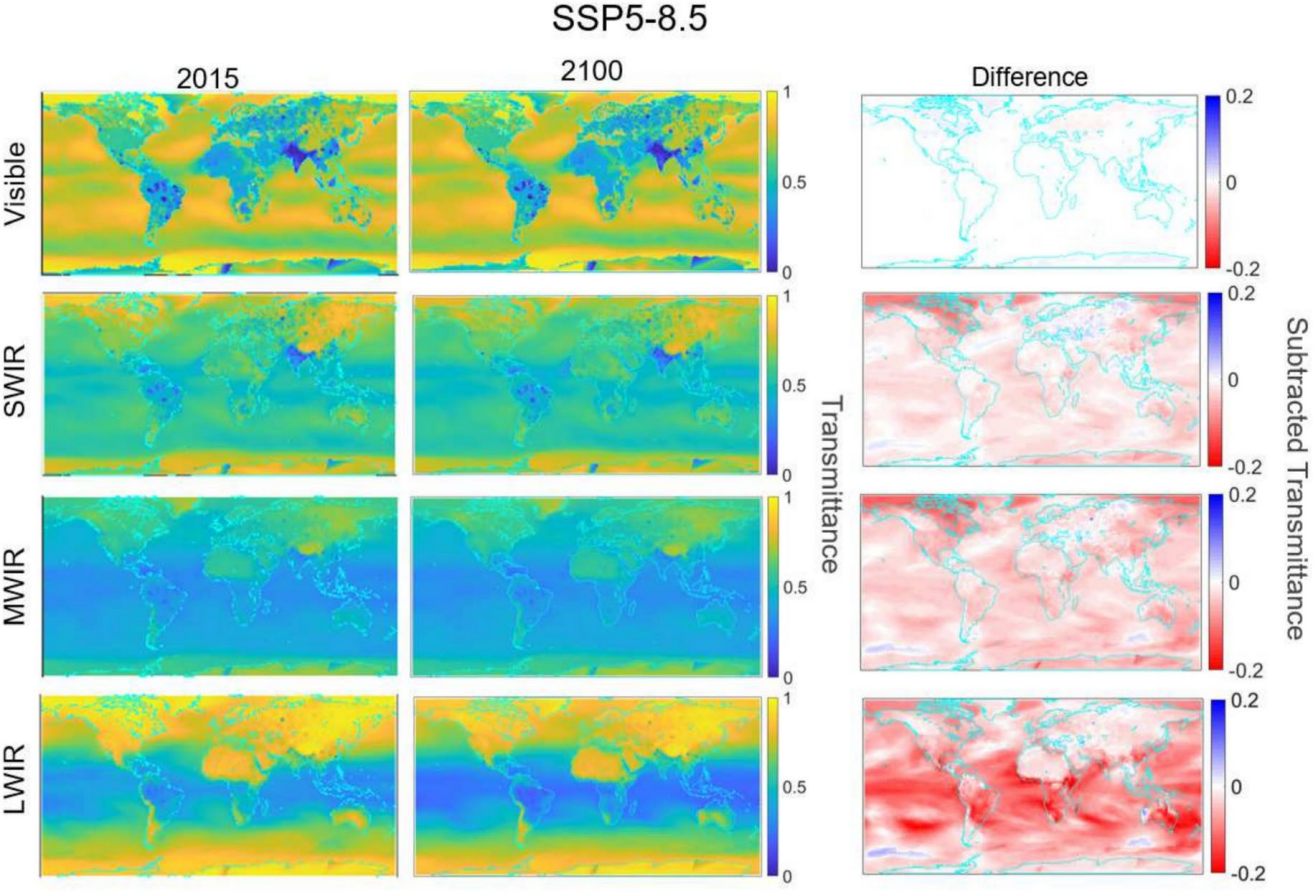
Global atmospheric transmittance over a surface 2 km horizontal path in 2015 and 2100 for the four EO/IR bands with seeded data from SSP5-8.5 (worst case climate scenario). Third column is subtracted transmittance (second column minus first column).

Global atmospheric transmittance analysis along a 2 km horizontal path calculated via MODTRAN was performed under CMIP6’s “best case” SSP1-2.6 and “worst case” SSP5-8.5 scenario at time steps of every other January from 2015-2100. This analysis showed very little drop in transmittance in the SSP1-2.6 case. This is expected, as no extreme meteorological changes were observed in the inputs. For SSP5-8.5 however, the reduction in transmittance is noticeably larger. Figure 6 shows these differences; note the drop in expected transmittance is most pronounced in the LWIR. This is due to significantly higher specific humidity levels, as water absorbs strongly in the LWIR. Also note that little change is observed in the visible/SWIR, as we have opted to assume no change in aerosol loading for this analysis.

**Fig. 7:**
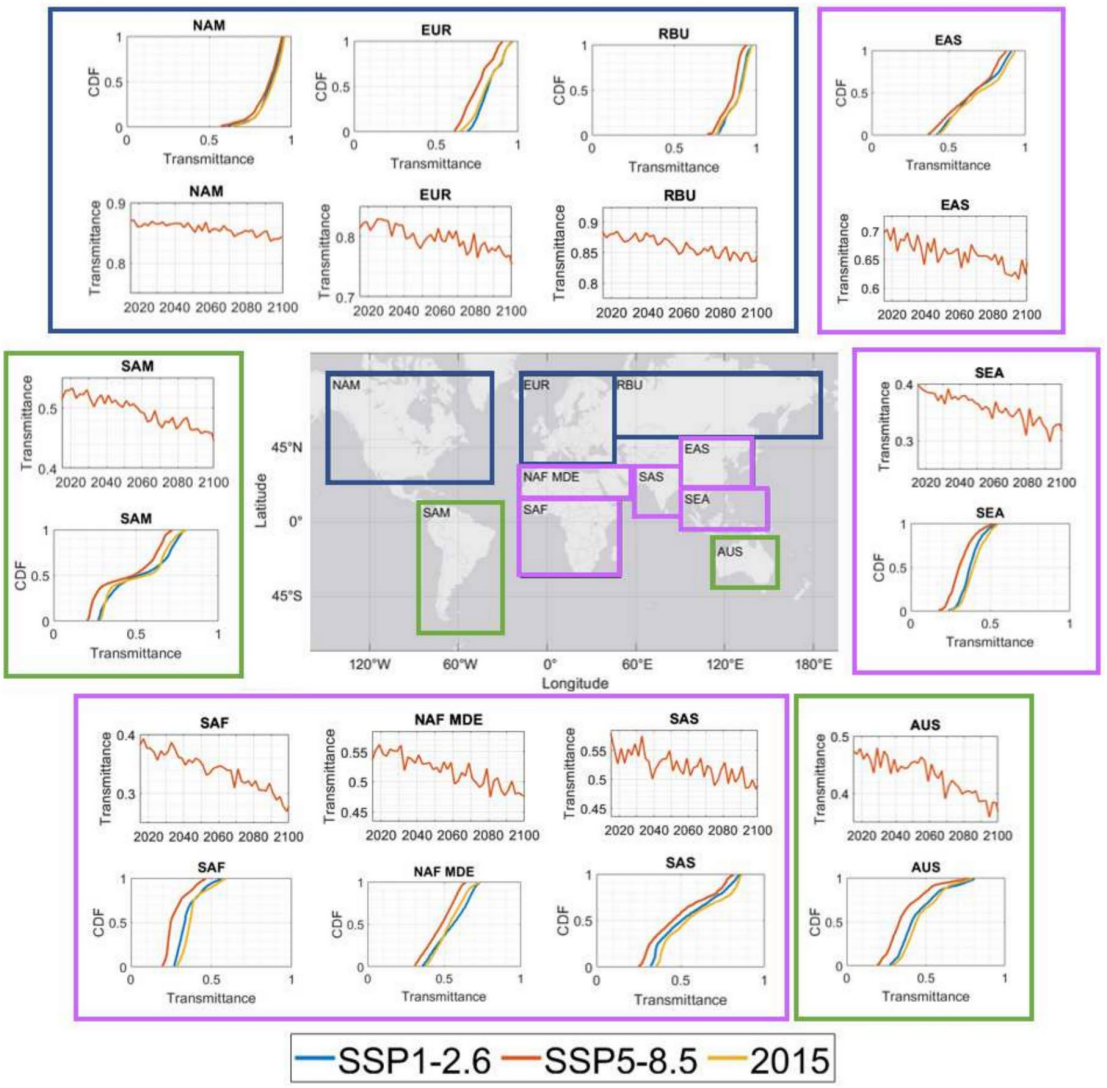
LWIR transmittance over a 2 km horizontal path averaged over 10 global regions from 2015-2100 for SSP5-8.5 and accompanying cumulative distribution functions (CDFs) of SSP1-2.6 and SSP5-8.5 in 2100, along with the reference year of 2015. These regions are: North America [NAM], South America [SAM], Europe [EUR], North Africa/Middle East [NAF MDE], South Asia [SAS], Australia [AUS], South East Asia [SEA], East Asia [EAS], South Africa [SAF], and Russia [RBU]. Colored frames represent grouped regions (blue=northern hemisphere high latitudes, purple = equatorial, green=southern hemisphere high latitudes).

Figure 7 shows the time evolution of the LWIR [8.0–12.0 $\mu$m] transmittance on this same 2 km path in terms of spatial averages over ten global regions of interest: North America [NAM], South America [SAM], Europe [EUR], North Africa/Middle East [NAF MDE], South Asia [SAS], Australia [AUS], South East Asia [SEA], East Asia [EAS], South Africa [SAF], and Russia [RBU]^54^. Note the global decrease of around 10% transmittance (for our path length of only 2 km), which tends to be worse around the equator and better at higher latitudes. This is again driven by increases in specific humidity which is higher in the equatorial regions due to higher average air temperatures, meaning the air can hold more water and more evaporation occurs^6^.

### Results - humidity correlation

**Fig. 8:**
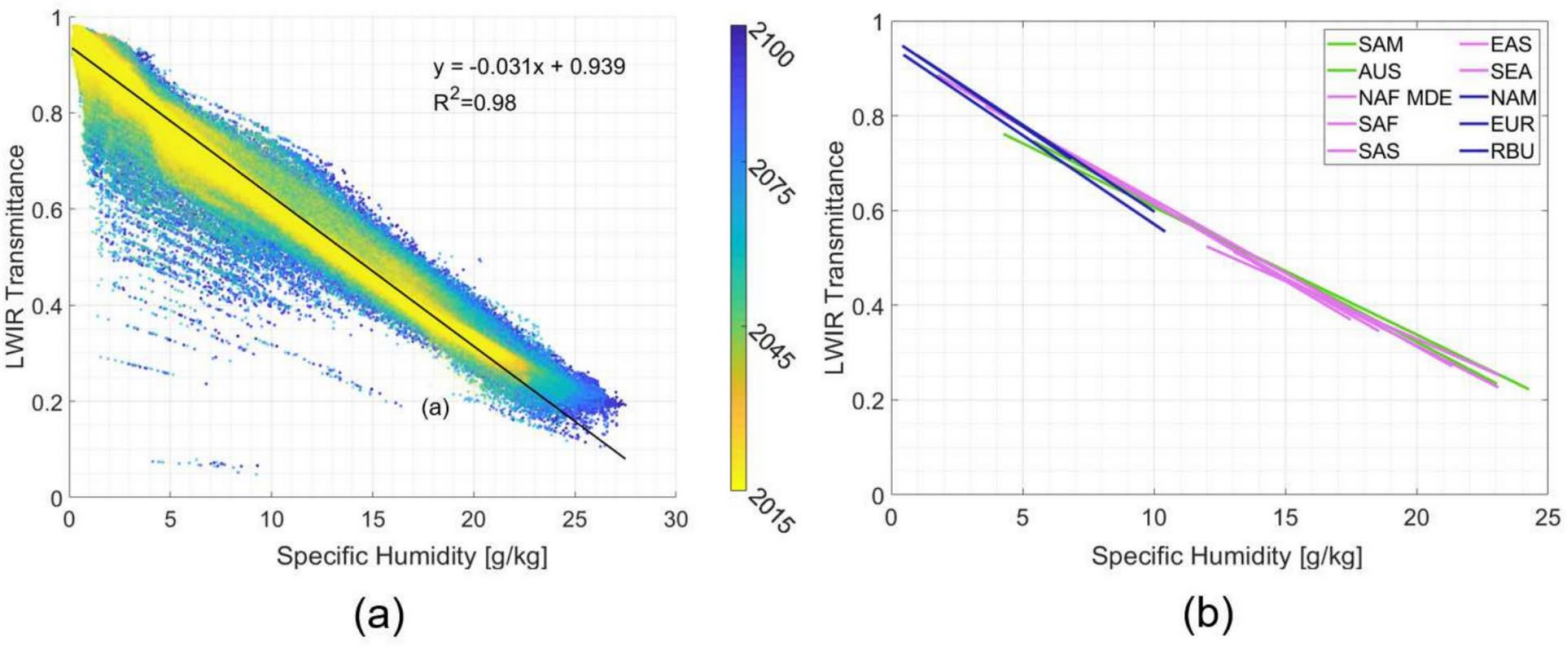
(**a**) Band averaged LWIR transmittance for all locations plotted in aggregate, with global linear fit and strong correlation $R^2=0.98$ and (**b**) Linear fits for band averaged LWIR transmittance for each of the 10 regions plotted in Figure 7.

In aggregating decades worth of atmospheric and corresponding MODTRAN data, it is possible to examine the correlation between specific humidity and atmospheric transmittance along this particular line-of-sight geometry in the LWIR. This data is shown in fig 8a aggregated across all locations, broken out by year. Note the strong, negative linear correlation in all locations, with years closer to 2100 pushing more towards higher specific humidity and therefore lower transmittances. Also note the strong $R^2$ correlation coefficient (0.98), suggesting that along a 2 km horizontal path knowledge of the specific humidity can directly give you the band averaged atmospheric transmittance in the LWIR.

In fig 8b, linear fits for each region are plotted on top of one another, with dryer/colder regions (North America, Europe, Russia) closer to the top left of the chart, and more warmer/more humid regions (Australia, South East Asia, South Africa) towards the bottom right.

### Results - band averaged extinction

Taking a broader time perspective and comparing January 2015 to January 2100, we have shown in Figure 6 an up to 20% decrease in the long wave infrared atmospheric transmittance for a path length as short as 2 kilometers - longer paths will see an even larger disparity. In order to evaluate the atmospheric degradation in a path agnostic way we calculate a first order approximation of the losses by determining a band averaged extinction coefficient $\sigma$ from the band averaged transmittance (Equation 1).

1 $$\begin{aligned} \begin{aligned} T = e^{-\sigma R} \\\ \sigma = \frac{-ln(T)}{R} \end{aligned} \end{aligned}$$

where *T* is the transmittance, $\sigma$ is the equivalent band average extinction coefficient [$km^{-1}$] and R is the range [km], in our case 2 km.

**Fig. 9:**
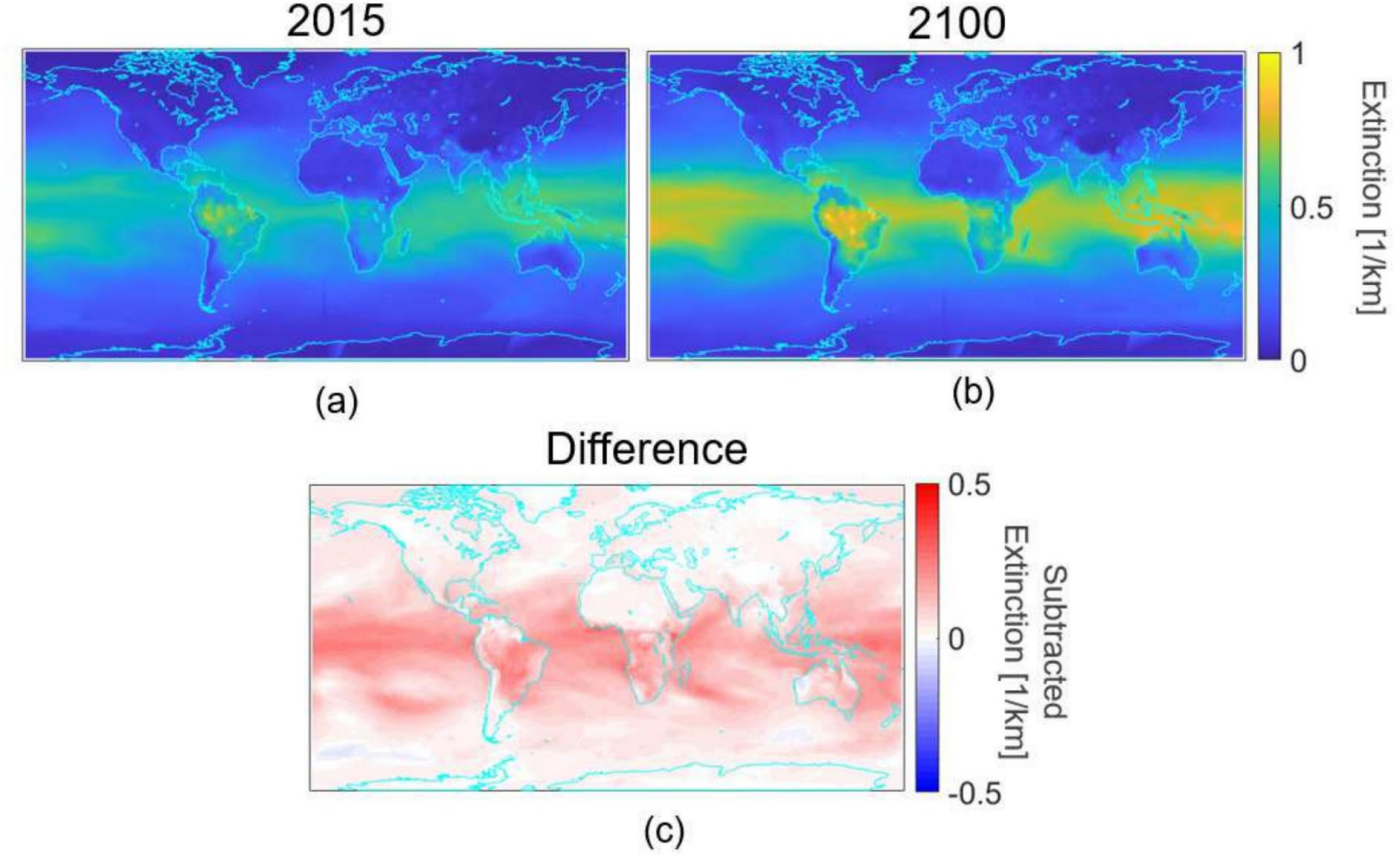
Band averaged extinction in the LWIR derived from transmittance values over a 2 km horizontal path.

Applying this methodology to the global long-wave infrared transmittance maps for a 2 km path we can determine band averaged extinction coefficients extracted from the appropriate MODTRAN run. The global map view for 2015 and 2100 are shown below in Figure 9. An increase in extinction values of 0.35 $km^{-1}$ in 2015 to greater than 0.7 $km^{-1}$in 2100 is seen in the equatorial regions. To put this into perspective, if this increase in extinction occurred in the human visual range it is the equivalent of reducing the visibility^37^ from 11 km in 2015 to a mere 5.5 km in 2100, comparable to a hazy day.

## Conclusions

An analysis of atmospheric transmittance via MODTRAN seeded with data from CMIP6 from the years 2015 through 2100 reveal increasingly challenging conditions for EO/IR propagation, particularly in the Long-Wave Infrared (LWIR) band due to increases in atmospheric water vapor. Current technologies that rely on EO/IR imagery for real-time evaluations will find challenges in maintaining dynamic range and contrast as transmittance is reduced in future years. In particular many EO/IR sensors are currently used to measure and understand the impacts of climate change itself and we will find ourselves more heavily relying on them to produce accurate data in areas such as crop health monitoring and food security, power-line inspections, and forest fire detection. This reduction in transmittance is even more pronounced under the worst-case scenario climate trajectory (SSP5-8.5) where the LWIR transmittance drops by as much as $20\%$ for a path length of only 2 km. This work could be extended in the future to longer and/or slant paths for aforementioned applications and many more. Analysis of the “best case” climate trajectory (SSP1-2.6) shows relatively stable conditions and little drop in transmittance, suggesting the future of EO/IR sensing will be dependent on the rate of advancement and adoption of emission curbing technologies and policies which drive our climate’s trajectory.

## Data Availability

The datasets analyzed during the current study are available in the ESGF repository, https://aims2.llnl.gov/search/cmip6.

## References

[1] NOAA-NCEI-ISD, “Global hourly - integrated surface database.” Available at https://www.ncei.noaa.gov/products/land-based-station/integrated-surface-database (2022/03/01).

[2] N. G. S. F. Center, “Aeronet - aerosol robotic network.” Available at https://aeronet.gsfc.nasa.gov/ (2023/08/01).

[3] NASA, “Calipso - data user’s guide.” Available at https://www-calipso.larc.nasa.gov/resources/calipso_users_guide/data_summaries/ (2022/03/01).

[4] *ERA5: data documentation*. Accessed: 2022-09-13.

[5] NASA, “Global modeling and assimilation office - merra2.” Available at https://gmao.gsfc.nasa.gov/reanalysis/MERRA-2/ (2022/03/01).

[6] I. P. on Climate Change, *AR6 - Synthesis Report Climate Change 2023* (2023).

[7] Wang, K., Dickson, R. & Liang, S. Clear sky visibility has decreased over land globally from 1973 to 2007. *Science* **323**, 1468–1470 (2009). 19286553 10.1126/science.1167549

[8] Nolte, C., Dolwick, N., Horowitz, L., *et al.*, “2018: Air quality. in impacts, risks and adaptation in the united states: Fourth national climate assessment,” (2018).

[9] Malm, W. et al. Spatial and seasonal trends in particle concentration and optical extinction in the united states. *Journal of Geophysical Research* **99**(D1), 1347–1370 (1994).

[10] Torseth, K. et al. Introduction to the european monitoring and evaluation programme (emep) and observed atmospheric composition change during 1972–2009. *Atmos. Chem. Phys* **1**, 5447–5481 (2012).

[11] D. O. of the Undersecretary for Policy (Strategy Plans and Capabilities), “Department of defense climate risk analysis. report submitted to national security council,” (2021).

[12] Haslebacher, C., Demory, M.-E., Demory, B.-O., *et al.* “Impact of climate change on site characteristics of eight major astronomical observatories using high-resolution global climate projections until 2050,” *Astronomy and Astrophysics* **1** (2022).

[13] Cantalloube, F. et al. The impact of climate change on atronomical observations0. *Nature Astronomy* **1**, 826–829 (2020).

[14] Karmakar, P. et al. Crop monitoring by multimodal remote sensing: A review. *Remote Sensing Applications: Society and Environment* **33**, 101093 (2024).

[15] Parihar, G., Saha, S. & Giri, L. I. Application of infrared thermography for irrigation scheduling of horticulture plants. *Smart Agricultural Technology* **1**, 100021 (2021).

[16] Hafeez, A. et al. Implementation of drone technology for farm monitoring and pesticide spraying: A review. *Information Processing in Agriculture* **10**(2), 192–203 (2023).

[17] Perryman, W. L. et al. Diel variation in migration rates of eastern pacific gray whales measured with thermal imaging sensors. *Marine Mammal Science* **15**(2), 426–445 (1999).

[18] Zitterbart, D. P. et al. Scaling the laws of thermal imaging-based whale detection. *Journal of Atmospheric and Oceanic Technology* **37**(5), 807–824 (2020).

[19] Smith, H. R. et al. A field comparison of marine mammal detections via visual, acoustic, and infrared (ir) imaging methods offshore atlantic canada. *Marine Pollution Bulletin* **154**, 111026 (2020). 32174485 10.1016/j.marpolbul.2020.111026

[20] Schroeder, W. et al. Integrated active fire retrievals and biomass burning emissions using complementary near-coincident ground, airborne and spaceborne sensor data. *Remote Sensing of Environment* **140**, 719–730 (2014).

[21] Hua, L. & Shao, G. The progress of operational forest fire monitoring with infrared remote sensing. *Journal of Forestry Research* **28**, 215–229 (2017).

[22] Salem, F., Kafatos, M., El-Ghazawi, T., *et al.* “Hyperspectral image analysis for oil spill detection,” *Summaries of NASA/JPL Airborne Earth Science Workshop* , 5–9 (2001).

[23] De Kerf, T. et al. A dataset of drone-captured, segmented images for oil spill detection in port environments. *Scientific Data* **11**, 1180 (2024). 39477957 10.1038/s41597-024-03993-8 PMC11525993

[24] Iwasaki, Y., Misumi, M. & Nakamiya, T. Robust vehicle detection under various environmental conditions using an infrared thermal camera and its application to road traffic flow monitoring. *Sensors* **13**(6), 7756–7773 (2013). 23774988 10.3390/s130607756 PMC3715224

[25] Farooq, M. A. et al. Object detection in thermal spectrum for advanced driver-assistance systems (adas). *IEEE Access* **9**, 156465–156481 (2021).

[26] Krišto, M., Ivasic-Kos, M. & Pobar, M. Thermal object detection in difficult weather conditions using yolo. *IEEE Access* **8**, 125459–125476 (2020).

[27] He, S., Yang, D., Li, W., *et al.* “Detection and fault diagnosis of power transmission line in infrared image,” in *2015 IEEE International Conference on Cyber Technology in Automation, Control, and Intelligent Systems (CYBER)*, 431–435 (2015).

[28] Ukiwe, E. K. et al. Deep learning model for detection of hotspots using infrared thermographic images of electrical installations. *Journal of Electrical Systems and Information Technology* **11**, 24 (2024).

[29] Berk, A., Anderson, G., Acharya, P., *et al.**MODTRAN 5.2.1 USER’s MANUAL* (2011). SSI and AFRL.

[30] Eyring, V. et al. Overview of the coupled model intercomparison project phase 6 (cmip6) experimental design and organization. *Geoscientific Model Development* **9**(5), 1937–1958 (2016).

[31] Jakob, C., Gettelman, A. & Pitman, A. The need to operationalize climate modelling. *Nature Climate Change* **13**, 1158–1160 (2023).

[32] Willitsford, A., Coye, P., Trop, C., *et al.* “Statistical approach to electro-optic and infrared transmission within the atmosphere through empirical cumulative distributions via modeling, simulation, and data on a global scale,” *Journal of Applied Remote Sensing* **17**(3) (2023).

[33] O’Neill, B. C. et al. The scenario model intercomparison project (scenariomip) for cmip6. *Geoscientific Model Development* **9**(9), 3461–3482 (2016). 10.5194/gmd-9-4521-2016 PMC5911933 29697697

[34] Riahi, K. et al. The shared socioeconomic pathways and their energy, land use, and greenhouse gas emissions implications: An overview. *Global Environmental Change* **42**, 153–168 (2017).

[35] Taylor, K., Stouffer, R. & Meehl, G. An overview of cmip5 and the experiment design. *Bulletin of the American Meteorological Society* **1**, 485–498 (2012).

[36] Stotts, L. & Schroeder, J. *Atmospheric Modeling Using PcModWin/MODTRAN*, SPIE PRESS (2019).

[37] Koschmieder, H. Theorie der horizontalen sichtweite. *Phys. Freien Atmos.* **12**, 171–181 (1924).

[38] Organization, W. M. “Guide to meterological instruments and methods of observation,” (2008).

[39] Wang, Z., Lin, L., Xu, Y., *et al.* “Incorrect asian aerosols affecting the attribution and projection of regional climate change in cmip6 models,” *npj Climate and Atmospheric Science* **2** (2021).

[40] Fu, W., Liu, Q., K. van den Bosch, C., *et al.* “Long-term atmospheric visiblity trends and their realtions to socieoeconomic factors in xiamen city, china,” *International Journal of Environmental Research and Public Health* **15** (2018).

[41] Kok, J. et al. Mineral dust aerosol impacts on global climate and climate change. *Nature Reviews earth and environment* **4**, 71–86 (2023).

[42] Chen, X. et al. Effects of human activities and climate change on the reduction of visibility in beijing over the past 36 years. *Environmental International* **116**, 92–100 (2018). 10.1016/j.envint.2018.04.009 29660613

[43] Gomez, J., Allen, R. J., Turnock, S. T., *et al.* “The projected future degradation in air quality is caused by more abundant natural aerosols in a warmer world,” *Communication’s earth and environment* **4**(22) (2023).

[44] Li, H. et al. Projected aerosol changes driven by emissions and climate change using a machine learning method. *Environmental Science and Technology* **56**, 3884–3893 (2022). 35294173 10.1021/acs.est.1c04380

[45] Li, C., Donkelaar, A. v., Hammer, M. S., *et al.* “Reversal of trends in global fine particulate matter air pollution,” *Nature Communications* **1** (2023). 10.1038/s41467-023-41086-z PMC10475088 37660164

[46] Myhre, G., Myhre, C., Samset, B., *et al.* “Aerosols and their relation to global climate and climate sensitivity,” *Nature Education Knowledge* **4** (2013).

[47] Kalisoras, A. et al. Decomposing the effective radiative forcing of anthropogenic aerosols based on cmip6 earth system models. *Atmospheric Chemistry and Physics* **24**(13), 7837–7872 (2024).

[48] Ramachandran, S., Rupakheti, M. & Cherian, R. Insights into recent aerosol trends over asia from observations and cmip6 simulations. *Science of The Total Environment* **807**, 150756 (2022). 34619211 10.1016/j.scitotenv.2021.150756

[49] Kunchala, R. K., Attada, R., Karumuri, R. K., *et al.* “Climatology, trends, and future projections of aerosol optical depth over the middle east and north africa region in cmip6 models,” *Frontiers in Climate* **6** (2024).

[50] Jaisankar, B., Tumuluru, V. L. K. & Anandan, N. R. Spatio-temporal correspondence of aerosol optical depth between cmip6 simulations and modis retrievals over india. *Environmental Science and Pollution Research* **31**, 16899–16914 (2024). 38329666 10.1007/s11356-024-32314-0

[51] C. for International Earth Science Information Network CIESIN Columbia University, “Gridded population of the world, version 4 (gpwv4): Population density.”

[52] Observatory, N. E. “Desert sample location map.” Available at https://earthobservatory.nasa.gov/biome/mapdesert.php (2023/06/01).

[53] Yukimoto, S., Kawai, H., Koshiro, T., *et al.* “The meteorological research institute earth system model version 2.0, mri-esm2.0: Description and basic evaluation of the physical component,” (2019).

[54] Lund, M. T., Myhre, G. & Samset, B. H. Anthropogenic aerosol forcing under the shared socioeconomic pathways. *Atmospheric Chemistry and Physics* **19**(22), 13827–13839 (2019).

